# Islet Transplantation: Current Limitations and Challenges for Successful Outcomes

**DOI:** 10.3390/cells13211783

**Published:** 2024-10-28

**Authors:** Allan Langlois, Michel Pinget, Laurence Kessler, Karim Bouzakri

**Affiliations:** 1 UR «Diabète et Thérapeutiques», Centre Européen d’Étude du Diabète, Université de Strasbourg, Boulevard René Leriche, 67200 Strasbourg, France; a.langlois@ceed-diabete.org (A.L.); m.pinget@ceed-diabete.org (M.P.); 2 Department of Endocrinology, Diabetes and Nutrition, University Hospital of Strasbourg, 67200 Strasbourg, France; laurence.kessler@chru-strasbourg.fr; 3 Inserm UMR 1260, Nanomédicine Regenerative, University of Strasbourg, 67085 Strasbourg, France

**Keywords:** islet transplantation, ischemia, inflammation, ER stress, mitochondria, immunosuppression, IBMIR, revascularization, transdifferentiation, dedifferentiation

## Abstract

Islet transplantation is a promising approach for treating patients with unstable T1DM. However, it is confronted with numerous obstacles throughout the various stages of the transplantation procedure. Significant progress has been made over the last 25 years in understanding the mechanisms behind the loss of functional islet mass and in developing protective strategies. Nevertheless, at present, two to three pancreases are still needed to treat a single patient, which limits the maximal number of patients who can benefit from islet transplantation. Thus, this publication provides an overview of recent scientific findings on the various issues affecting islet transplantation. Specifically, we will focus on the understanding of the mechanisms involved and the strategies developed to alleviate these problems from the isolation stage to the post-transplantation phase. Finally, we hope that this review will highlight new avenues of action, enabling us to propose pancreatic islet transplantation to a maximum number of patients with T1DM.

## 1. Introduction

Type 1 diabetes mellitus (T1DM), or insulin-dependent diabetes, is an autoimmune disease characterized by the dysfunction of T lymphocytes, which are responsible for destroying the β-cells of the pancreas. As a result, insulin production becomes insufficient or non-existent, leading to prolonged hyperglycemia [1], and the main treatment for this consists of subcutaneous injections of insulin several times a day. However, this strategy is sometimes insufficient to effectively regulate glycemia and necessitates alternative therapies. One of these alternatives is pancreatic islet transplantation. This minimally invasive strategy is indicated for unstable T1DM patients suffering from recurrent hypoglycemia leading impaired awareness of hypoglycemia, and who do not respond to intensive medical treatment. It is also performed after kidney transplantation in cases of end-stage renal failure [2,3,4]. Current clinical data show that islet transplantation is a sound therapeutic option for patients with T1DM, as it stabilizes glucose metabolism, normalizes glycemia, and considerably reduces episodes of severe hypoglycemia [2,3,5]. In addition, the complications of diabetes are delayed or attenuated and are associated with an improvement in diabetes-related quality of life [2,3,5,6]. Furthermore, a recent clinical study demonstrated the beneficial effect of islet transplantation on kidney graft survival in T1DM patients [7]. Moreover, islet transplantation has recently been shown to improve cognitive impairment associated with glycemic imbalance [8].

Pancreatic islet transplantation involves several stages. It begins with the removal of a pancreas from a brain-dead donor, which is then rapidly transferred to the cell therapy unit to isolate the islets. A collagenase solution is injected into the pancreas via the Wirsung duct, and digestion is carried out in a Ricordi chamber under mechanical agitation. The islets are then purified using density gradient centrifugation of the digesta. At the end of this procedure, the islet preparation is assessed in terms of purity, viability, sterility, number, and sometimes for functionality [9]. Current recommendations for clinical perfusion are at least 3500 Islet Equivalent Quantity (IEQ)/kg of recipient body weight (with a total of at least 10,000 IEQ/Kg for a complete transplant procedure), with viability greater than 80% and purity greater than 50% if possible [10]. Nevertheless, this consensus may be modified in countries where the procedure is now covered by the healthcare system. Currently, it is well known that the main objective of islet transplantation is not necessarily to achieve insulin independence. Indeed, the current outcome for optimal graft function are an HbA1c <7.0% without severe hypoglycemia, a >50% reduction in insulin requirements, and the restoration of clinically significant C-peptide [11,12]. Nonetheless, these goals remain difficult to achieve as islet transplantation encounters numerous obstacles throughout the various stages of transplantation, resulting in a significant loss of viable and functional pancreatic β-cell mass. Indeed, the quantity of functional pancreatic islets continues to decrease throughout the entire process, from the donor stage to the post-transplant period. According to the literature, this can be explained by the induction of cell death mechanisms, by inflammatory reactions, and by hypoxia induced before and after transplantation [13,14,15,16,17]. This is also due to host immune rejection reactions, the undesirable effects of the immunosuppression used to combat them [18], and the recurrence of post-transplant autoimmunity [19,20]. As a result, it takes an average of two to three pancreases to treat one patient, making it difficult to offer islet transplantation to all patients with T1DM who require it.

To cope with the excessive number of pancreases required for one patient, it is crucial to protect and preserve the functional mass of pancreatic islets from the earliest stages of transplantation. Therefore, it is important to understand how these deleterious phenomena affect islet number and the function and survival of pancreatic β-cells in order to develop graft protection strategies. With that in mind, this manuscript presents an overview of the detrimental mechanisms affecting both the quality of pancreatic islets for transplantation and the survival and function of engrafted islets. Specifically, we highlight here the importance of the extracellular matrix (ECM) for optimal β-cell function and survival. We then examine the deleterious effects of ischemia, with a focus on endoplasmic reticulum (ER) stress and mitochondrial dysfunction. Furthermore, we address the post-transplantation problem of rejection and the role of immunosuppression, Instant Blood-Mediated Inflammatory Reaction (IBMIR), islet revascularization, and peri-transplant insulin management. We also explore the potential role of cellular plasticity in graft efficacy and seek to present examples of solutions to address all of these issues. Finally, we hope that this review will give insight into new avenues for action, allowing islet transplantation to be offered to a larger number of patients with T1DM.

## 2. Quality of Islet Preparation for Transplantation: Where Do We Stand?

**Figure 1:**
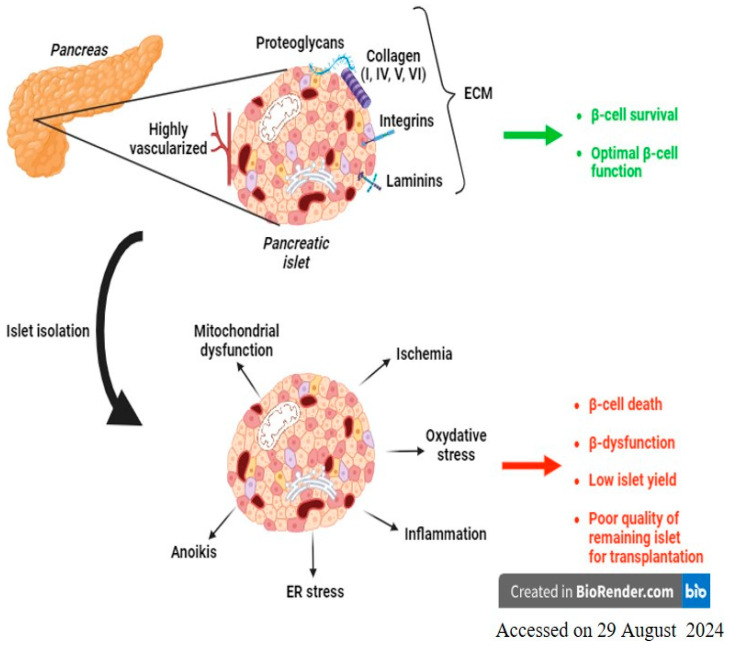
Mechanisms affecting the pancreatic islet during the isolation process. During the isolation process, pancreatic islets are removed from their natural environment, which is essential to their survival and function. The extracellular matrix (ECM) and vascularization are key elements. Their alteration will induce cell death mechanisms, β-cell dysfunction, low post-isolation yield, and poor quality of the islets to be transplanted.

One of the main challenges in reducing the number of pancreases to be used for a single patient is to improve post-isolation islet yields while preserving their viability and function. However, the efficiency and quality of islet isolation depends on the age of the donor, his or her body mass index (BMI), the composition of the pancreatic ECM, and the effect of its destruction during the digestion phase. Another critical factor to consider in protecting islet preparation is the ischemia faced by the pancreas and pancreatic islets following the disruption of the blood vessel network [21,22]. In this part of the review, we have chosen to focus on the ECM and ischemia problematics in order to improve the quality of pancreatic islets intended for transplantation (Figure 1).

### 2.1. Importance of the ECM for Isolation Effectiveness and β-Cell Function

The main component of the pancreatic islet ECM is collagen (Types I, IV, V, and VI), making it the key element to target for optimizing the efficiency of pancreas digestion and increasing the final yield of the islet. In this context, Meier R et al. [21] recently found that the efficiency of collagen digestion was, as expected, donor dependent but not significantly correlated with age, gender, BMI, warm or cold ischemia time, and pancreas weight. Furthermore, the authors estimated that the correlation between the degree of collagen digestion and a “good” or a “bad” islet isolation is between 60 and 80%. However, no link was found between collagen content or collagen type (predominance of type VI in older donors) and islet digestion levels. Finally, based on their results, the authors proposed that a pancreatic collagen digestion rate of 60% should be considered the threshold above which islet isolation is likely to be successful and transplantable. However, beyond the ultimate goal of maximizing the amount of islets released during digestion, it is crucial to protect the ECM, as its destruction causes the loss of β-cell survival and function. Indeed, the ECM is fundamental to the normal endocrine functions of pancreatic islet cells, and its destruction during enzymatic digestion causes β-cell death due to anoikis and impairs insulin secretion [23]. It is widely recognized that each collagen type plays an important role in the proliferation, survival, function, and differentiation of pancreatic cells [24]. The islet ECM is also composed of laminins, such as laminin-511, which has been found to be essential for β-cell proliferation and insulin transcription [25]. Additionally, integrins, which are other components of the ECM, contribute to the loss of function of β-cells after the isolation process. Indeed, integrin-mediated adhesion signaling in response to the pancreatic ECM plays crucial roles in β-cell survival and insulin secretion [23]. Arous C et al. [26] recently demonstrated, in primary rat β cells and mouse insulinomas, the existence of cooperation between different AKT isoforms and focal adhesion kinase (FAK)-dependent adhesion signaling, which controls insulin secretion or prevents it under stressful conditions. In particular, the authors showed that β-cells form adhesions containing integrins. These then provide an anchor to the pancreatic ECM and initiate intracellular signaling via FAK and paxillin. Finally, in view of their overall findings, the authors suggested that adhesion-mediated signaling may explain β-cell dysfunction. Along these lines, other studies have reported that β1-Integrin-A is a key player in the control of pancreatic β-cell insulin secretion via interaction with SNARE proteins, a crucial complex involved in insulin granule exocytosis [27,28].

Therefore, the islets obtained for transplantation are weakened and devoid of the ECM, contributing to the loss of functional mass of the islets from the first days after transplantation. Indeed, after intraportal injection, there is a long delay before the islets are implanted in the recipient’s liver, particularly during which the ECM is recovered [29,30]. During this time, many islets die or lose their function due to the alteration of molecular signaling pathways induced by ECM components and, in part, due to anoikis, as described above. Chronologically, the injected islets do not have a basal membrane, and, for the first 5 days after transplantation, there are few or no links between the islet and liver’s ECM. These connections take around 14 days to develop and another two weeks for the complete remodeling of the ECM. Therefore, it takes approximately one month after islet injection to restore homeostasis [29].

To summarize, important strategies to optimize the islet isolation step, to increase post-digestion islet yield, and to preserve functional islet mass include protecting or restoring the ECM after enzymatic digestion and/or promoting islet–ECM interactions post-transplant. Particularly, this will require a deeper understanding of the composition of the ECM, the precise interactions between its various components, and the mechanisms involved in their activation. On the one hand, several approaches were developed either to protect the ECM pharmacologically by targeting the mechanisms mentioned above—for example, to reestablish a β-cell–ECM interaction—or by using ECM components. To this end, Santini-Gonzalez J et al. [31] established a method to construct a peri-islet ECM on the surfaces of isolated rat and human islets by a co-assembly from a solution of laminin, nidogen, and collagen IV proteins. Interestingly, the authors found that although freshly isolated human islets and those coated with their matrix showed similar glucose-stimulated insulin secretion (GSIS) after 24 h of culture, the coated islets exhibited a stronger insulin secretion response to high glucose after seven days compared to uncoated islets. This opens the prospect of extending the time between the end of the isolation procedure and transplantation without compromising the functional β-cell mass. However, although the results presented in this study are highly relevant and respond to a real need for a solution to the current problem, no in vivo validation has been performed to confirm the effectiveness of this strategy. Another proposed approach is to supplement the culture medium with islet basement membrane components [24,32]. Brandhorst D et al. [32] showed that an individual supplement of collagen IV, laminin-521, and Nidogen-1 in a culture medium improved the survival and function of human islets. Nidogen-1 showed the greatest effect. However, these benefits were lost when the components were combined, possibly due to the increased production of chemokines and reactive oxygen species. According to the authors, this might have been a concentration problem. More recently, the same team tested Perlecan as a solution to improve the functional and morphological survival of islets prior to and after transplantation [33]. The authors compared the effect of collagen IV, Laminin-521, and Perlecan individually or in combination on human islet survival, function, inflammation, and morphological integrity. Notably, while each component had beneficial effects, Perlecan exhibited the most potent anti-inflammatory and anti-apoptotic effects, thus making it a promising strategy for preserving the functional mass of pancreatic islets pre- and post-grafting. However, this finding also requires in vivo confirmation. Finally, pharmacological approaches have shown their feasibility for restoring the islet ECM. For example, Cardoso LEM et al. [34] found that semaglutide, a GLP-1 receptor agonist, allowed for islet ECM remodeling in a diet-induced obese mouse model. In particular, the authors showed that this molecule allowed for the renewal of the ECM components of islets such as heparan sulfate proteoglycans, hyaluronane, chondroitin, and collagen. Thus, they suggested that semaglutide could restore a healthy functional environment in pancreatic islets.

### 2.2. Ischemia: The Dark Side of Islet Isolation Success

Vascularization is essential for the survival and optimal function of pancreatic islets. As a matter of fact, they are highly vascularized, receiving 6–20% of the direct arterial blood flow, which ensures adequate gas and nutrient exchange, as well as waste removal. It also allows for the rapid delivery of insulin into the bloodstream in response to rising glucose levels [35]. Moreover, the ECM from islet microcapillary endothelial cells affects β-cell spreading and enhances insulin secretion [36]. Consequently, interruption of the blood or oxygen supply due to the isolation process has serious and immediate consequences on the metabolic and morphological integrity of islets [37,38,39]. Indeed, it has been shown that the number of functional islets available for transplantation correlates with ischemic time [40,41]. In particular, ischemia/reperfusion (I/R) phenomena have been identified as major factors involved in the significant loss of the functional mass of pancreatic islets during isolation [22,42]. During this process, pancreatic islets face different episodes of ischemia, starting from the donor stage. Indeed, the pancreas are either acquired from cadaveric donors that have suffered warm ischemia of up to 35 min with periods of ventilation [43] or from donors that have undergone resuscitation of up to 30 min [44]. In addition, after the retrieval of the pancreas, they undergo a period of cold ischemia, which is the time between pancreatectomy and collagenase perfusion, and this period plays a role in the variability in islet isolation outcomes [40,45]. Therefore, this time must be as short as possible and ideally less than 12 h to obtain excellent results [45,46]. Indeed, through a retrospective study conducted on 452 pancreas isolations, Wassmer CH et al. [45] showed a significant decrease in isolation success, defined as an islet yield ≥200,000 IEQ, after 8 h of total ischemia time, 7 h of cold ischemia time, and 80 min of organ removal time. Nevertheless, a 50% probability of successful isolation has been determined for a total duration of ischemia of more than 12 h, although this is donor dependent. The islets are then isolated through enzymatic digestion from the pancreas at 37 °C; cellular metabolism is reactivated. In addition, the exocrine tissue is susceptible to ischemia and releases deleterious factors during the isolation stage [47]. Once dissociated from the pancreas, the islets are exposed to the atmospheric, partial oxygen pressure of 765 mmHg against 40–60 mmHg that is usually found in the pancreas, mimicking reperfusion around the islets. From a mechanistic point of view, during ischemia, anaerobic respiration takes over for the maintenance of cell survival [48]. This type of respiration increases the presence of H^+^ ions in the intracellular medium and is accompanied by an increase in the concentration of calcium ions. All of this is combined with low ATP production and the destruction of the adenosine reserve necessary for ATP recycling. In addition, when the pancreas and islets are re-oxygenated, they suffer significant damage. Indeed, the lack of ATP, the imbalance of calcium ions, and the reactive oxygen species produced by the flow of oxygen and glucose activate oxidative stress and inflammation phenomena that cause apoptosis, cell necrosis, and β-cell dysfunction [48,49]. Usually, ER stress and mitochondrial dysfunction appear to be important responses to inflammation and oxidative stress that impact islet survival and function [50,51,52,53].

## 3. ER Stress Has Side Effects on Pancreatic β-Cells

ER is an essential organelle that intervenes in particular in the synthesis, modification, quality control, and trafficking of proteins [54,55,56]. In the case of ER functional perturbations, it leads to what is known as ER stress, characterized by the accumulation of unfolded or misfolded proteins in the ER lumen, resulting in cell dysfunction [57]. Moreover, ER stress has been widely shown to be involved in islet dysfunction across the diabetes spectrum, as both ER trafficking and protein-folding capacity are integral to β-cell protein quality control and insulin synthesis [58,59]. In addition, ER is an important storage site for calcium and plays a crucial role in regulating insulin release [60]. This implies that ER is closely related to insulin secretion, as Ca^2+^ is a key factor involved in the canonical mechanisms of insulin secretion [61]. Consequently, any disruption to the proper functioning of ER would have a deleterious effect on the mechanisms of insulin secretion. Indeed, many studies have shown that defects of ER integrity are associated with the defective trafficking of pro-insulin and a reduced number of insulin granules in pancreatic β-cells. Furthermore, it is well known that excessive ER stress can lead to β-cells apoptosis and dysfunction [58,59,62,63]. In response to ER stress, the unfolded protein response (UPR) is activated to restore ER homeostasis. This notably allows for the maintenance of integrity and normal β-cell function and is classically controlled by three canonical ER resident transmembrane proteins, activation factor 6 (ATF6), inositol-requiring enzyme 1, and endoplasmic eIF2a kinase PKR/pancreatic (PERK/PEK) [56,57]. Each then produces a transcription factor (ATF4, XBP1s, ATF6, respectively), triggering the activation of ER stress-inducible gene transcription, whose function is to increase the functional capacity of the organelle and decrease the arrival of newly synthesized proteins [55,64,65,66]. Interestingly, Omori K et al. [67] demonstrated, by electron micrographs, that prolonged cold ischemia induced severe swelling and the disruption of cell and mitochondrial membranes, as well as an expanded ER in isolated β-cells. The authors also found that the process of isolating islets, independently of the cold ischemia of the pancreas, activated the UPR, whereas the BiP protective chaperon was even more overregulated by cold ischemia/pancreas warming. Therefore, this response participates in the death of pancreatic islets by apoptosis and necrosis. Thus, limiting ER stress would be a relevant target to limit the loss of pancreatic islets and increase the functional β-cell mass available for transplantation. In this sense, recent studies have shown that pancreatic β-cells were sensitive to excessive ER stress and dysregulated eIF2α phosphorylation. The authors suggested that this discovery should be taken into account when designing new therapeutic approaches for diabetes [57]. Furthermore, proinflammatory cytokines, such as interleukin (IL)-1β and Interferon-γ, induce β-cell ER stress and contribute to pancreatic β-cell loss in T1DM [50]. These data also highlight the importance of limiting I/R phenomena to reduce inflammatory reactions and ER stress in pancreatic β-cells. This would protect the islets from apoptosis and preserve their function. Finally, several studies have demonstrated the distinct or combined role of ER stress and mitochondrial dysfunction in β-cell functionality [53,58].

## 4. Mitochondrial Dysfunction in Pancreatic β-Cells Leads to Impaired Insulin Secretion and Apoptosis

Currently, mitochondrial oxidative metabolism appears to be an important bioenergetic contributor to the triggering of insulin secretion by glucose, thanks to its capacity to generate 36 ATP per oxidized glucose molecule [68]. Indeed, ATP is a pivotal actor of the canonical mechanism of insulin secretion, leading to the closure of ATP-dependent potassium channels, which in turn promotes the opening of Ca^2+^ potential channels in response to cell membrane depolarization. The resulting increase in cytosolic Ca^2+^ concentration then triggers the exocytosis of insulin granules [61,69,70,71,72]. Mitochondrial Ca^2+^ plays also a central role in GSIS. Indeed, in a model of rat islet β-cells expressing mitoS100G (calcium-binding protein G), Wiederkehr A et al. [73] demonstrated that a mitochondrial matrix Ca^2+^ signal is required for the potentiation of glucose-induced second-phase insulin secretion. More recently, Georgiadou E et al. [74] showed in β-cell-specific *Mcu*-KO mice that the mitochondrial Ca^2+^ importer (MCU) complex is crucial for mitochondrial Ca^2+^ uptake in pancreatic β-cells and is required for normal GSIS. Mitochondrial dynamics, ensured by processes of fusion, fission, and transport of mitochondria, are also important for insulin secretion and β-survival [75]. It was recently shown that the inhibition of Dynamin-related protein 1 (Drp1), GTPase protein, using small hairpin Drp1 RNAs in INS1a cells and pancreatic islets, decreased mitochondrial fusion, mitochondrial membrane potential, ATP production, and insulin secretion [75,76]. Moreover, its knockdown in NIT1 β-cell lines impaired GSIS [75,77]. These mechanisms are crucial for the proper functioning of cells because they allow for the elimination of defective mitochondria through a specific process of autophagy, called mitophagy [78]. Furthermore, it is widely established that insulin secretion, β-cell death, and mitochondrial dysfunction are closely associated with good mitochondrial quality control through mitophagy. Recent findings have shown that the loss of mitochondrial quality control in pancreatic β-cells leads to the accumulation of dysfunctional mitochondria, which causes oxidative stress due to excessive production of reactive oxygen species (ROS), β-cell dysfunction, and apoptosis [51,52,75,79]. Moreover, Aoyagi K et al. [72] demonstrated in a β-cell-specific mitophagy reporter mouse model that hypoxia, induced by a high-fat diet, triggered the aberrant accumulation of dysfunctional mitochondria (due to excessive ROS production) and the defective maintenance of mitochondrial function and impaired insulin secretion. However, the authors also reported that these side effects of metabolic stress occurred despite an increase in the mitophagy process. These data confirm, first, its huge role in cellular defense, that it could be a good indicator of islet functional mass, and, finally, that it represents a potential target of interest to protect islets. Nevertheless, it also seems that targeting this mechanism alone would certainly not be sufficient to protect pancreatic islets, which suggests the necessity to act upstream, reducing negative mitochondrial function modulators such as inflammation and oxidative stress widely induced, notably, by ischemia.

Recent work has focused on Imeglimin and has shown that this molecule exerts favorable effects on pancreatic β-cells by improving morphology in mitochondria, increasing the number of insulin granules and insulin secretion [52]. Imeglimin is a tetrahydrotriazine-containing class of oral glucose-lowering agents synthesized from Metformin, which reduces glucose and lipid biosynthesis in the liver by suppressing anabolic processes and increases glucose uptake in skeletal muscle [80]. It was also known to normalize glucose tolerance and improve insulin sensitivity by protecting mitochondrial function from oxidative stress and favoring lipid oxidation in the livers of mice fed a high-fat, high-sucrose diet [80,81]. The favorable effect of Imeglimin is so convincing that a phase 3 clinical evaluation is underway as part of the treatment of Type 2 Diabetes Mellitus (T2DM) [82]. In particular, recent papers showed that Imeglimin treatment also improved mitochondrial morphology and suppressed apoptosis in β-cells in diabetic model db/db mice [83,84]. Moreover, this molecule improves β-cell apoptosis by modulating the ER homeostasis pathway. Indeed, Li J et al. [80] showed in an Akita mouse model that Imeglimin upregulated the expression of ER-related molecules, including Chop (Ddit3), Gadd34 (Ppp1r15a), Atf3, and Sdf2l1, and it decreased eIF2α phosphorylation after treatment with thapsigargin and restored global protein synthesis in β-cells under ER stress. These data altogether indicate that Imeglimin could represent an interesting avenue to prevent the loss of the functional mass of pancreatic islets to be transplanted by reducing ER stress and mitigating the accumulation of dysfunctional mitochondria through mitophagy. Finally, another interesting avenue to target in order to improve mitochondrial function and protect β-cell function is that of microRNAs (miRNAs), which are small noncoding RNAs implicated in the regulation of gene expression [85]. Moreover, they play a pivotal role in β-cells, as these are involved in endocrine cell differentiation [86], in the regulation of β-cell mass and development [87], in insulin secretion mechanism [88], and in apoptosis [89]. For example, Krishnan P et al. [90] recently demonstrated that miR-146a-5p is upregulated in pancreatic islets treated with proinflammatory cytokines and that its overexpression leads to mitochondrial dysfunction and apoptosis in β-cells. Interestingly, the authors were able to reverse these deleterious effects by inhibiting miR-146a-5p.

## 5. The Limits of Islet Transplantation: Where Do We Stand?

**Figure 2:**
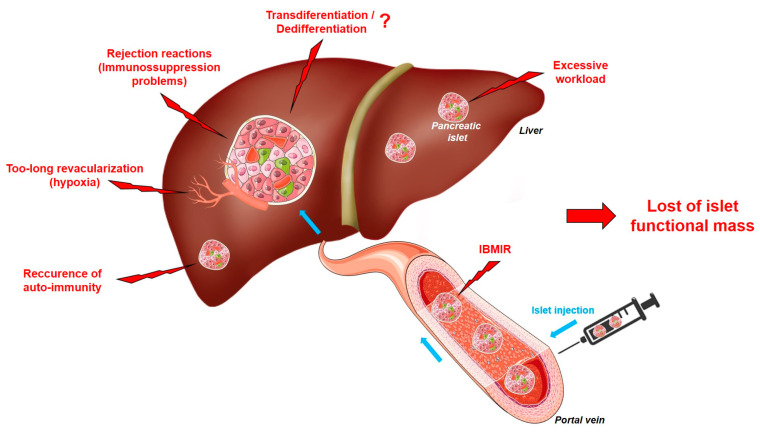
Mechanisms affecting pancreatic islet after transplantation. Immediately after injection, at least 50% of pancreatic islets will be destroyed by IBMIR, in connection with the activation of coagulation mechanisms. The functional mass of pancreatic islets will further decrease drastically due, on the one hand, to rejection reactions and their immunosuppression strategies and, on the other hand, by the late implementation of functional vascularization. Moreover, islet implantation in its new environment is accompanied by significant cellular stress, causing oxidative stress and inflammation, leading to cell death and loss of graft efficacy. Finally, the question arises as to whether cellular plasticity, which may occur within the islets, is involved in the loss of beta cell function.

As we have just described, the pancreatic islets proposed for transplantation already suffer greatly during the isolation procedure. Unfortunately, they will also be confronted with other deleterious mechanisms, which will significantly reduce the functional islet mass from the very first moments post-injection. In this second part of this review, we will therefore look at these issues, including rejection reactions, IBMIR, islet revascularization, and peri-transplant insulin management. Finally, we will discuss the possible existence of cellular plasticity within the islets that could occur after transplantation and could have an impact on graft function (Figure 2).

### 5.1. Islet Transplantation and Immunosuppression

One of the main reasons for islet cell death and loss of functional β-cell mass post-transplantation is the alloimmune reaction [10,91]. After islet intraportal injection, antigens derived from the graft, such as insulin, IA-2, glutamic acid decarboxylase 65 (GAD65), and ZnT8, activate dendritic cells (DCs) and macrophages that subsequently activate Cluster of Differentiation (CD) 8 T cells, CD4 T cells, macrophages, and neutrophils, leading to islet destruction. CD4 and CD8 T cells are also activated by DCs derived from the islet graft and by B cells [19,92]. Added to these mechanisms, engrafted islets face recurrence of the recipient’s autoimmunity. Indeed, memory autoimmune cells (CD4 T cells, CD8 T cells, and DCs) are rapidly reactivated after islet transplantation, and they expand and destroy the islet graft [19,20].

Currently, glucocorticoid-free immunosuppression is proposed for transplanted patients. The protocol includes an induction phase at each islet infusion, followed by a maintenance phase during the entire lifespan of the islet transplant [10]. However, there are no established treatment protocols at present. The Edmonton protocol, which proved effective, proposes an immune-suppressive treatment composed of an anti-IL-2-receptor antibody (a T-cell activation inhibitor) before each islet infusion, combined with sirolimus, a mammalian Target Of Rapamycin (mTOR) inhibitor, and low-dose tacrolimus (a calcineurin inhibitor). However, β-cell toxicity of tacrolimus has been demonstrated, which is due to its inhibitory effect on the mTOR pathway [93]. More recently, Perrier Q et al. [94] found that Tacrolimus impaired insulin secretion without affecting cell viability in Min6 cells and rat islets. Interestingly, they demonstrated that Belatacept, a costimulation blocker, preserved both insulin secretion ability and cell viability as compared to Tacrolimus. Furthermore, Belatacept use in kidney transplant recipients led to improvement in HbA1c in cases of post-transplant diabetes or de novo diabetes after transplantation [95]. These promising data make this treatment an interesting immunosuppressive strategy for pancreatic islet transplantation. Other new drugs and new combinations have been proposed in recent years, such as T-cell depleting agents (teplizumab…), lymphocytetracking inhibitors (efalizumab…), anti-inflammatory agents that inhibit Tumor Necrosis Factor (TNF)-α (etanercept…), or IL-1β (anakinra…) [10]. Moreover, Lemos JRN et al. [96] recently analyzed the effect of GAD65 and IA2 autoantibody status on graft survival and the attainment of insulin independence in subjects with T1DM who underwent islet transplantation. Patients with persistent single anti-GAD65 antibody positivity had a shorter graft survival period than subjects negative for anti-islet autoantibodies (GAD65/IA2). Nevertheless, graft survival time was no different in subjects with single IA2 positivity. Then, Alibashe-Ahmed M et al. [97] demonstrated on a non-obese diabetic (NOD) mouse model, that Toll-like receptor 4 (TLR4) signaling contributes to the development and maintenance of autoimmune diabetes. Moreover, the authors found that the cyclohexene derivative, CLI-095, an inhibitor of TLR4 signaling, could be part of a preventive strategy targeting patients at risk for type 1 diabetes. In addition, it is well described that the alteration of regulatory T cells, such as CD3^+^, CD4^+^, CD25^+^, CD127^low^, and FOXP3^+^ (Treg) cells, is implicated in the onset of autoimmune diseases and the progression of T1DM, whose function is to help control or prevent these pathologies [98,99]. Thus, increasing Treg function has emerged as a strategy to reduce or eliminate systemic immunosuppression after islet transplantation in preclinical and clinical studies. For example, Cabello-Kindelan et al. [100] evaluated Treg engraftment and therapeutic efficacy in a non-obese diabetic (NOD) mouse model of autoimmune diabetes using nonablative, combinatorial regimens involving the anti-CD3 (αCD3), cyclophosphamide, and IAC (IL-2/JES6-1) antibody complex. Interestingly, they found that this novel combinatorial therapy promotes the engraftment of autoantigen-specific donor Tregs and controls islet autoimmunity without long-term immunosuppression. More recently, Wu X et al. [99] evaluated the chimeric antigen receptor (CAR) targeting of human Tregs to monocytes, a human β-cell line, and human islet β-cells in vitro. Interestingly, the authors showed that CD39 expression segregated CAR Treg cytotoxicity, and CD39^+^ Tregs represent a safer choice for CAR Treg therapies targeting tissues for tolerance induction. In addition, remarkable progress has been made in recent years on the immuno-isolation of pancreatic islets by encapsulation. Indeed, a number of medical devices have been able to demonstrate their effectiveness, such as the Beta-O_2_ device [101,102], which consists of a container with a PTFE semi-permeable membrane inside, where islets are embedded in an alginate hydrogel. This device allowed for maintaining graft function up to 10 months but with low levels of circulating C-peptide and with no impact on metabolic control [103]. One drawback to this system is the need to fill it with O_2_ every day, which can be a constraint for the patient. Apart from this, islet nano-encapsulation could also be a promising option for conferring immunological protection to the islet. Moreover, thanks to an ultra-thin layer of encapsulating material, this approach allows for easy oxygen and nutrient inflow and insulin outflow [103,104]. Another advantage of this approach would be the possibility to inject encapsulated islets into the liver and benefit from the first-pass effect. However, further studies on efficacy and safety are still needed. Finally, the future treatment of rejection reactions may lie in vaccination. Indeed, negative vaccination is currently proposed as an original approach to promote transplant tolerance. This strategy consists of the delivery of a donor antigen before or after transplantation, together with a “negative adjuvant” to selectively inhibit the alloimmune response [105].

Although considerable progress has been made in recent years in terms of treatments to effectively combat rejection reactions, it is clear that another major challenge remains to be overcome in protecting the functional mass of transplanted islets—that of providing an early and effective diagnosis of their onset. Currently, allograft rejection is diagnosed by tissue biopsy, and treatment depends on the type and severity of rejection identified by the biopsy [106,107]. However, this approach remains difficult to apply for islet transplantation, as islets are dispersed in the liver post-injection, making it difficult to detect them and to have enough islets to obtain a representative biopsy. Furthermore, this technique is too invasive to be practiced and may lead to complications. Interestingly, from transplanted patients with suspected rejection episodes (SREs), Lanstra CP et al. [91] showed for the first time the important features of SREs, including incidence and timing after transplantation. Thus, they demonstrated that if an SRE was left untreated or identified too late, islet graft function was almost completely lost. Moreover, the authors proposed that unexplained hyperglycemia, increased insulin requirements, and the abrupt decrease in C-peptide are markers indicating the sudden loss of islet graft function, and they can contribute to the diagnosis of islet rejection. Nevertheless, this proposal, although very pertinent, needs to be confirmed, given the small number of patients involved in this study. Other biomarkers of interest for post-injection graft quality were also studied in the bloodstream, such as insulin messenger RNA, miRNA375, and unmethylated insulin DNA [108,109,110]. Interestingly, they increase within 24 h of transplantation, and some are associated with worse functional outcomes from the islet graft. This suggests that they may be useful in predicting early grafting and evaluating interventions to improve the survival of the islets during the grafting period [111]. Finally, exosomes, as carriers of major complex molecules of donor histocompatibility, are a candidate biomarker of rejection that are processed by the cells that present the recipient antigen and are detected by the immune cells of the recipient [112].

### 5.2. IBMIR

Approximately 50% of islets are destroyed immediately after intraportal transplantation by an IBMIR induced by the activation of coagulation, complement, and proinflammatory pathways such as Nuclear Factor-kappa B (NF-kB) [20,113]. Briefly, during intraportal injection, islets are exposed to blood, and immunoglobulins -G and -M bind to the islet surface, inducing complement activation and the deposition of C3-fragments (C3b/iC3b). After that, the Tissue Factor released by islets interacts with factor VIIa, which induces activation of the coagulation system. The thrombin generated activates platelets and initiates fibrin formation. Platelet activation increases the affinity of integrins (GPIIb-IIa and α2ß1) for fibrin and collagen, respectively, and promotes the binding of activated platelets to the islet surface. In addition, platelet activation induces complement activation and leads to the generation of C3a and C5a anaphylatoxins, which recruit and activate monocytes and granulocytes near the islets. Platelet activation induces thrombin release, generating fibrin, which eventually forms a capsule around the islets trapped by platelets, granulocytes, and monocytes. Through the action of chemotactic factors, released directly from the islets (e.g., IL-8, MIF, MCP-1, soluble CD40, TNF-α…) or generated by complement activation (C5a), granulocytes and monocytes infiltrate the islets, leading to islet cell destruction [20,114]. Recently, it was demonstrated that thrombin–antithrombin complexes and platelet activation occurred in the first 12 h post-islet injection. Natural killer (NK) cells and macrophages also contribute to IBMIR [20,115].

To limit IBMIR, some treatments were applied as NF-kB inhibitors or anticoagulants such as low-molecular-weight dextran sulfate, heparin treatment, or etanercept (TNF-α inhibitor) [116,117,118]. Islet preparations are supplemented with heparin (70U/kg of the recipient’s body weight), followed by an intravenous infusion of heparin for 2 days and the subcutaneous administration of heparin for 7 days after intraportal injection of the islets. [2]. Recently, Wang J et al. [119] found that α-1 antitrypsin efficiently suppressed IBMIR and enhanced islet engraftment by the mitigation of coagulation and suppression of cytokine-induced JNK and NF-kB activation. Moreover, Turan A et al. [113,120] proposed an original new approach, which consists of modifying the islet surface to overcome early graft loss triggered by IBMIR. Indeed, the authors generated a chimeric form of thrombomodulin (SA-TM), a multifaceted innate immune modulator, with streptavidin for transient display on the surfaces of islets modified with biotin. Interestingly, their strategy enhanced engraftment and sustained survival in a minimal mass mouse intraportal islet transplantation model. Finally, to reinforce the effectiveness of their approach, the same team demonstrated that islets can be coengineered with SA-TM and SA-CD47 molecules as a combinatorial approach to improve engraftment by inhibiting IBMIR.

### 5.3. Islet Revascularization

As previously described, transplanted islets are avascular, and the rapid restoration of blood flow seems to be vital to preserve functional β-cell mass. However, this process takes several days to a few weeks [121], and the delayed functional formation of micro vascularization deprives islet cells of oxygen and nutrients, causing their apoptosis and/or necrosis [29,36,122]. Indeed, vascular buds form on the periphery of the islets within 14 days of transplantation, but the core of the islet is still poorly vascularized during this period. It takes about two more weeks for the core to revascularize and oxygen levels and waste disposal to recover. Finally, the transplanted islets only come close to normal levels of endogenous oxygen after one month post-injection [29]. It is therefore essential to develop effective strategies to optimize this revascularization, and this must involve a good understanding of the mechanisms involved. It is widely documented that angiogenesis is the main mechanism involved in restoring islet vascularization [121]. However, this process requires several days to weeks, and delayed functional microvasculature formation starves islet cells of oxygen and nutrients, causing their apoptosis and/or necrosis [122,123]. More recently, the fundamental role that the endothelial cell can play on the pancreatic β-cell was highlighted. Particularly, many advances have been made in recent years concerning the existence of close communication between these two cell types, which influences their survival and function [35,124,125]. For example, Daniel B et al. [36] demonstrated that glucose-induced insulin secretion is modulated by an effective paracrine interaction between islets’ microcapillary endothelial cells and β-cells. Moreover, Figliolini et al. [126] found that biologically active islet-derived extravesicules can shuttle anti-apoptotic and pro-angiogenic mRNAs and miRNAs into endothelial cells. The idea of the importance of cross-communication strategies was developed. Indeed, Kado T et al. [127] recently found that the co-transplantation islet/skeletal myoblast cells could be an effective way to improve islet revascularization. In a model of islet transplantation under the kidney capsule of streptozotocin-induced diabetic mice, the authors found that the co-injection of an islet with a myoblast improved significantly glycemia regulation. They also demonstrated that the myoblast allowed for an increase in the insulin-positive area and the number of microvessels compared to islets alone. Interestingly, in vitro, they showed that the myoblast secreted pro-angiogenic cytokines such as Vascular Endothelial Growth Factor (VEGF), Hepatocyte Growth Factor (HGF), and stromal-derived factor-1α. This therefore makes them targets of interest to potentiate islet revascularization. Indeed, it is well known that a defect in VEGF-A signaling can impair β-cell proliferation, insulin secretion, and glucose homeostasis [35,124,128]. Moreover, the HGF produced regulates β-cell proliferation and is essential for β-cell differentiation, function, and proliferation [35,124]. Thus, the team classified this impact on islets as an “indirect effect”. Nevertheless, they also suggested the “direct effects” of the myoblast due to IL-6 secretion on enhanced islet graft function via JAK-STAT signal activation [127]. These data are very interesting, because it was recently demonstrated that endothelial cells can promote the function of internal β-cells in pseudo-islets through the BTC-EGFR-JAK/STAT signaling pathway [129]. This reinforces the notion of close communication between the β-cell and the endothelial cell and the importance of rapid and adequate revascularization of the transplanted islet for its survival and function. Moreover, the interaction between the myoblast and islets may effectively activate the production of key factors and consequently enhance the beneficial effects observed. Given the current knowledge about the beneficial effects of cross-cell communication involving a variety of factors, co-transplantation appears to be a promising approach for improving efficiency in islet transplants. Nevertheless, further research is needed on the safety of co-transplantation, as there are still many questions to be answered regarding the fate and medium- or long-term effect of undifferentiated cells on the patient, on the graft, and on the function of the organ receiving the cell preparation. In view of these questions that remain pending, we can also question their possible use in the liver due to the risk of hemorrhage related to intraportal injection, which may lead to the spread of undifferentiated cells throughout the body. Finally, another approach consists of the generation of pre-vascularized islet organoids. Indeed, Wassmer CH et al. [45] recently generated and transplanted pre-vascularized insulin-secreting organoids composed of rat islet cells, human amniotic epithelial cells, and human umbilical vein endothelial cells. They then demonstrated in diabetic mice that these organoids have better function than native islets in vitro, better engraftment, and better revascularization compared with conventional islet transplantation. Interestingly, the authors found that these beneficial effects are mainly due to the interaction between the cells forming the organoids, resulting in up-regulation of genes promoting angiogenesis (*VEGF-A*) and β-cell function (*glp-1r*, *pdx1*). In conclusion, this latest study once again confirms the importance of cellular crosstalk for optimal islet function and that organoids could be a highly promising β-cell replacement therapy.

### 5.4. Insulin Is Needed After Transplantation to Protect Islets from Their New Environment

Immediately after injection, the newly isolated pancreatic islets, weakened by all the constraints described above, find themselves in a new hyperglycemic environment, where the demand for insulin to regulate glycemia is high. It is well known that prolonged or repeated exposure to high glucose concentrations has deleterious effects on the expressions of genes related to insulin production, insulin content (GSIS), and β-cell survival [120,121,122]. Furthermore, as previously mentioned, islet transplantation can take several weeks to allow the islets to function properly due to the re-establishment of the ECM and vascularization. As a result, the islets very quickly find themselves faced with an excessive workload, leading to a loss of functional mass. To avoid this, blood glucose levels are rigorously controlled from the very first moments after transplantation, and intensive exogenous insulin therapy is introduced. This is then progressively reduced and withdrawn depending on the glycemia measured [130,131,132]. Moreover, insulin therapy is generally implemented when random glycemic sampling demonstrates, on three subsequent occasions within the same week, fasting values >140 mg/dL (7.8 mmol/L) and postprandial values >180 mg/dL (10.0 mmol/L) or after recording two consecutive A1c values >6.5% [130]. Kikawa K et al. [122] demonstrated in a model of islet transplantation under the kidney capsule of diabetic *Akita* mice that insulin therapy improved the outcome of islet transplantation, improved glucose tolerance, and reduced islet apoptosis.

### 5.5. Could the Efficacy of Islet Transplantation Be Affected by Cellular Transdifferentiation/Dedifferentiation?

Cellular transdifferentiation represents the modification of one cell type into another by exposure to different factors, without passing through the pluripotent cell stage, unlike induced Pluripotent Stem Cells [133]. Cell dedifferentiation is the process by which cells grow reversely from a partially or terminally differentiated stage to a less differentiated stage within their own lineage [134]. Interestingly, transdifferentiation is based on the principle of cellular plasticity, which plays a crucial role in glycemia homeostasis [135]. It is well described that pancreatic cells, such as ductal cells, centro-acinar cells, α cells, and δ cells, are able to transdifferentiate in functional β-cells to compensate for insulin secretion impairment to maintain normoglycemia [133,136]. It has also been shown that T2DM is characterized by a reduction in β-cell mass and an alteration in their function, associated with a failure to maintain their identity. This leads to dedifferentiation of β-cells into non-functional endocrine progenitor cells or transdifferentiation into other endocrine cell types [137,138,139]. Furthermore, β-cells fail to maintain a fully differentiated state of response to drugs in patients with diabetes with periods of poorly controlled and long-lasting hyperglycemia [137]. However, the molecular mechanisms are still elusive. Recently, Oger F et al. [139] suggested that the cell-cycle regulator and transcription factor E2F1 are critical for maintaining β-cell identity and function through the sustained control of β-cell and non–β-cell transcriptional programs. In particular, the authors showed in E2f1 β-cell-specific knockout isolated islets (*E2f1 ^β^*^−/−^), using genomic analysis, a loss of β-cell identity markers with a strong decrease in transcript levels of *Pdx1*, *Ins2*, *Pcsk9*, *Foxo1*, and *Glp1r*. Moreover, in these islets, they observed the upregulation of genes in *E2f1 ^β^*^−/−^ mostly associated with inflammatory response, including the hepatic fibrosis signaling pathway, iNOS signaling, and Toll-like receptor signaling. The down-regulated genes, on their part, revealed an enrichment of gene networks involved in the insulin secretion pathway or maturity-onset diabetes of young signaling. Finally, the pharmacological inhibition of E2f1 in Min6 cells and human islets impairs GSIS and alters β- and α-cell gene expression. The environment could also influence β-cell identity. It was reported that environmental factors induce reversible DNA modifications, which affect gene transcription and organ functions, such as islet function [140]. This is known as epigenic regulation, and its dysregulation leads to the development of metabolic disorders such as T2DM [141,142]. In the context of T2DM, Bornaque F et al. [141] suggest that environmental variations, such as glucose, control m^6^A methylation, one of the most epitranscriptomic modifications prevalent in eukaryotes [143], in pancreatic β-cells, playing a key role in the control of gene expression and pancreatic β-cell functions. Indeed, the authors found that high glucose concentrations decrease in mice and human islet m^6^A methylation, altering m^6^A demethylase gene expression. Moreover, m^6^A enzyme inhibition modulates the expression of genes involved in pancreatic β-cell identity and glucose-stimulated insulin secretion.

All these data suggest that post-transplant islets may also be affected, and it seems interesting to investigate the possible involvement of these phenomena in the loss of functional β-cell mass. Indeed, it is currently well accepted that reconstituting missing cell populations by promoting innate and adaptive cell plasticity in situ is a promising prospect for treating degenerative diseases [136], as well as diabetes, and hence could be another means of improving islet transplantation success. In this context, it has notably been proposed to use viral vectors to deliver β-cell transcription factors, such as Pdx1, MafA, Ngn3, or NeuroD1, to induce the pancreatic transdifferentiation of cells to replace the altered β-cell population [144]. However, even if these approaches are interesting, there is still the question of their feasibility post-transplant, as they seem so difficult to implement.

## 6. Conclusions

Throughout this article, we have aimed to provide an overview of recent findings on the deleterious mechanisms affecting pancreatic islet quality during the isolation process (Figure 1) and to present proposals to address these issues. Additionally, we discussed challenges (Figure 2) and potential solutions for transplanted islets. This allowed us to appreciate the numerous complications that pancreatic islets face during the various stages of transplantation. As a result, there is still a great deal of work to be done to protect the functional mass of islets and reduce the number of pancreases needed for a single patient. Moreover, the standardization of the complete procedure between the different centers remains one of the key issues to be solved in order to optimize the success of islet transplantation. However, the remarkable scientific advancements in the past few years in understanding these deleterious pathways have enabled the identification of numerous targets for action and the establishment of various strategies (genetic, pharmacological, organoid, encapsulation, and stem cell strategies), all of which are equally promising. Ultimately, these advances bring hope that more patients with T1DM will be treated in the near future.
